# Dental Ethics—Fee Bills—Tooth Powders and Mouth Washes—Their Uses and Abuses

**Published:** 1880-11

**Authors:** C. M. Wright


### THE DENTAL REGISTER.

VOL. XXXIV.] NOVEMBER, 1880. [No. 11.

### "DENTAL ETHICS—FEE BILLS—TOOTH POWDERS AND MOUTH WASHES—THEIR USES AND ABUSES."

READ BY C. M. WRIGHT, BEFORE THE AMERICAN DENTAL SOCIETY OF EUROPE, AT LUCERNE, AUGUST 30, 1880.

MR. PRESIDENT AND GENTLEMEN—I have taken up my pen a dozen or more times to write a short paper for your consideration, on a part, or the whole of the fifth subject, and after having dipped the pen into clean ink, spread out my paper and blotters, etc., squared myself to the table, as Sam Weller did when he wrote the immortal "Valentine" ending, "your love-sick Pickwick," I have not been able to get on any further than the heading, and "Mr. President and Gentlemen."

It has not been, however, may I assure you, from a lack of thoughts on the subject or subjects that I could not get on. No. No! Gentlemen, there has been no paucity of thoughts, important ideas and deep feelings on the subject; but either from too great a fullness, that prevents utterance, or like a bottle of beer suddenly turned over with the neck into a glass, the beer does not flow steadily but with hurried gulps and blubbers, or from an inability to marshal the thoughts, ideas and feelings into regular companies and platoons, I have been compelled to put away my pen, time and again, and wait another opportunity when my muse, or mood, or something, will allow me to make a better mount on the prose steed Pegasus. As the time of meeting of our Society has drawn nearer I have been compelled to jump on the back of this (in my case scrubby) pony, Pegasus and with a "hoopa-la," rush into the ring and let the ideas and feelings marshal themselves.

In regard to Dental Ethics there is no code that half expresses the laws that should fill the heart of every one of us. No society has succeeded in tabulating a distinct series or code of laws for our government. But every man should have, or has, some sort of undefined principles of what should be the conduct of himself and brother practitioner toward patients and each other. As this code has not been defined clearly every man has been allowed to decide for himself, and, consequently, in many minor details a great variety of opinions exists. The medical profession have been more decided in their laws, and the medical code even if not written out in all its details has precedent to back it, and is more generally understood than the Dental Code of Ethics.

If a dozen dentists were asked to define in one word the sum of Dental Ethics, they would, no doubt, each answer, *gentleman.* A gentleman is a personification of Dental Ethics. Gentlemen need no Code of Ethics. The perfect gentleman instinctively follows correct principles in his conduct toward patients even in trying times, and has no difficulty in treating his fellow practitioners properly. This is all true, and I, like the other eleven dentists, would answer the same. Practically, the word gentleman means —do as I do, and it is liable to have different shades of meaning quite dependent upon the coloring of the peculiar Ego. If Ego is an avaricious man, many little grasping, selfish acts would be quite compatible with his idea of the conduct of a gentleman.

To treat his patients absolutely according to the Golden Rule; to charge what Ego would be willing to pay; to do no more for them than Ego would be willing to do, if he was doing it for himself; to skip a prospective decay in a fissure, easy to prepare and not absolutely necessary, and thereby leaving the twenty franc gold piece snugly ensconced in the portmonnaie of the patient instead of slyly drawing it out of that resting place and depositing it into Ego's pocket. These things affect the avaricious Ego; and his idea of gentleman means in reality a polite thief, unamenable to the law of the land. He violates no written code. There could be no penalties attached to this dishonesty, and yet how many, many such dishonest acts are perpetrated in the quiet of our offices upon the public. How wrong this is; how injurious finally to the good name and standing of dentistry.

The jealous man colors his meaning of the one gentleman. The selfish man thinks he is a gentleman, when he believes that he has the right to honor, fame and the good opinion of his patients regardless of the honor, fame and good name of any other dentist. A shrug, a smile, a wagging head, from a man who would scorn to say a word against another dentist. How many times has the confidence of a patient in his former dentist been blasted by such simple means? How many of us have been guilty of a similar fault against what is known in our language as *native dentists*, even when we have purified our souls sufficiently to be able never to condemn a brother of our society? This is a selfishness inexcusable in a truly noble-minded professional gentleman. Every infirmity of the mind or temper, every petty vice of the heart can be displayed in the practice of profession, and in the dazzling light of a pure conscience, how many of us must hide our faces and cry mea culpa! Gentlemen, I am sorry I have not the pen and the tongue of an Evangelist at a camp meeting, for the subject is a worthy one, and I feel that if I had the power of a full use of these little implements I could so scorch the consciences of my beloved hearers that the mourners bench would be filled with penitents, and I should myself drop down among you and pray earnestly for more light in the rugged, crooked, and dark lane of our professional walk, that we may see better in the future how to walk erect, as *gentlemen*, in the full and noble sense of the word, untrammelled by our vices of constitution and training and made noble by a pure and strict sense of honor.

The next branch of the subject, *Dental Fees*, is one that I hope will be discussed at this meeting. It is a question of importance and a uniform opinion, a standard opinion by a majority of this society would be as useful as it would be interesting. Shall we receive fees for each operation and present our accounts to our patients as the hotel keepers do for each item, adding the charge for bougies and service; for a turn of the wrist, left side, five francs; for a twist of back, right side, ten francs; scaling a bit of tartar from the right dens sapientia, fifteen francs; one extra tartar for a friend of the patient, five francs, and so on; or, shall we emulate the cab drivers and arrange our fees on the hour plan. So much for the first hour; so much for every additional half hour, etc. Or shall we not rather present our accounts simply to professional services on this date for Mister, so much; to professional services on that date for Madam, so much, and leave the commercial aspect out altogether? The opinions of the society are earnestly requested. It is a mooted question, and the settlement of it at this meeting would help to distinctly define our standard among the liberal professions of the day. Personally, I began with the "bougie and service, and omnibus" style of fee bill, advanced, as I believed to the cab-drivers standard and am now trying to get fixed on the, what to me seems a higher plane of—"to professional services."

In referring to the last part of this mighty subject, "Tooth Powders and Mouth Washes, their uses and abuses," I do not think it especially interesting to discuss their uses and abuses. We have each of us talked every day for a dozen years, more or less, to our patients about the uses of tooth powders and mouth washes, and we have all seen the effects of the abuses of brushes and powders and have warned our patients against them. The part of the subject that I should like to have discussed by this society to-day, is, shall the dentist manufacture and sell powders, washes, or any other articles, such as brushes, soaps, tooth-picks, fine, satin-lined, silver-stoppered, ground glass, etc., toilet cases, fine tooth combs, sponges, hair pins and the like? Is it compatible with a high standard of professional respectability for the dentist to engage in trade to the extent that I have hinted at or to any other extent? Is he a high-toned professional gentleman, who, while exercising his skill in operating on living tissue of the human organization, occupies his tongue at the same time, or shortly after, in the "here's your nice cool lemonade, only five cents a glass," especially when he knows the ice in the lemonade is only glass? He may argue that lemonade is good for the patient and that if he don't sell his lemonade another will, and that the profits on the lemonade pays for the rent of his office, or he may take highegrounds, and with a noble earnestness of countenance assert that A. T. Stewart sold tape by the metre; or that commerce has studded our oceans with sails, sir, and bound our land with iron hoops. sir. Ha! Sneer at commerce, sir. Why, commerce is the friend of mankind, the regulator of the destinies of nations, sir; the foe of war, the friend of blessed peace.

This is all noble and true, but unfortunately is not the question, for it can be answered as easily, that the barber engages in commerce of the same sort, and remains a cringing, garlic-eating, sour-smelling barber still, and in ninety-nine cases in a hundred never becomes what he should be, an artist, or even a skilled artisan. But it is not the barber that we care to discuss now. Dentistry is a youth, so to speak, and we want to make him a gentleman We want to place him on a plane equal to that of the scientist, the physician and the surgeon. We want to elevate his professional standard, so that people may learn to respect the calling itself. So that no gentleman need blush when he is called upon to say, in any society, at any club of gentlemen, "sir, I am a dentist. And in order to raise this youth to this position, we must each personally strive to correct our habits, or avoid the appearance of anything that in the most fastidious society looks like unprofessional conduct, or like "Yer's yer nice cool lemonade, only five cents a glass."

The compounding of medicines is the apothecary's or chemist's duty. Let the apothecary and the chemist compound medicines and take care of his own social and professional standing. The selling of tooth brushes, tooth-picks, hair-brushes, fancy toilet articles, is a branch of trade followed by many very good people. Let these very good people make their fortunes and drive their tandems if they please, while we must walk. It is not our business and we degrade our profession and ourselves, besides violating a principle of modern commerce, in regard to specialties, when we try to pirate their profits. Let us be dentists and gentlemen, and keep out of other occupations while we call ourselves dentists, and make the name *dentist* a respectable one. Respectable, not in the sense that every honest calling is respectable, but in the higher sense that we shall be socially respected where we are not now.

Respectable in the sense that, to-day, if a gentleman in our ranks happens to visit the sea-side or a club, or a reception, he drops his title and is careful to be known only as Mr. Blank, tourist, visitor, gentleman.

Have I said enough ? Have I not dear brothers struck chords in your hearts that vibrate in harmony with a desire for a nobler position for our youth Dentistry? As god-fathers of this youth shall we not watch over him a little more carefully and while we purify ourselves may we not feel that we are doing our duty to ourselves, our children, and the future of dentistry. Let us discuss!

